# Regulation of inflammation by omega-3 and omega-6 fatty acids: a meta-analysis of randomized trials

**DOI:** 10.3389/fnut.2026.1799601

**Published:** 2026-05-05

**Authors:** Zicheng Huang, Xiangjun Zhan, Jun Jin, Qingzhe Jin

**Affiliations:** 1 State Key Laboratory of Food Science and Resources, School of Food Science and Technology, Jiangnan University, Wuxi, China; 2 Food Laboratory of Zhongyuan, Luohe, Henan, China; 3 Department of Food Science and Technology, School of Agriculture and Biology, Shanghai Jiao Tong University, Shanghai, China

**Keywords:** anti-inflammatory, meta-analysis, omega-3 fatty acids, omega-6 fatty acids, polyunsaturated fatty acids

## Abstract

This study quantitatively compared the anti-inflammatory effects of omega-3 and omega-6 polyunsaturated fatty acids (PUFAs) across different populations via meta-analysis. A systematic search of six major databases identified nine randomized controlled trials (RCTs) involving 504 participants. Data on inflammatory markers, including interleukin-6 (IL-6), C-reactive protein (CRP), interleukin-1 beta (IL-1β), and tumor necrosis factor alpha (TNF-α) were extracted, and subgroup analyses were performed based on fatty acid type, population health status, and intervention duration. Effect sizes were synthesized using random/fixed effect models. Overall, omega-3/6 supplementation showed no significant effects on IL-6, CRP, and TNF-α (*p* > 0.05). However, IL-1β levels were significantly reduced in the intervention group (MD = −0.04, 95% CI: −0.07 to −0.01, *p* = 0.02), with a more pronounced effect observed in the omega-6 subgroup (MD = −0.05, *p* = 0.03). No significant differences in IL-6 or TNF-α were observed between healthy individuals and patients following omega-3/6 intervention. Additionally, omega-3 supplementation did not improve IL-6 levels in Metabolic dysfunction-associated steatotic liver disease (MASLD) patients (MD = 0.00, *p* > 0.05). Short-term interventions (10–12 weeks) did not significantly alter IL-6 (omega-6) or TNF-α (omega-3) levels. These findings suggest that omega-6 PUFAs may inhibit IL-1β release via specific pathways, while both omega-3 and omega-6 exert limited effects on most inflammatory markers.

## 1 Introduction

Omega-3 fatty acids, a major class of long-chain polyunsaturated fatty acids, its derivatives primarily include eicosapentaenoic acid (EPA) and docosahexaenoic acid (DHA), which are naturally abundant in sardines and mackerel (1). As the human body cannot synthesize omega-3 fatty acids endogenously, dietary intake is essential. These fatty acids play vital roles in regulating inflammation, circulatory function, circulatory function, and nervous system activity (2). Their anti-inflammatory effects are largely mediated through the suppression of cellular inflammatory mediators (3). In addition, omega-3 fatty acids have demonstrated protective effects against cardiovascular disease. For example, Cao, et al. (4) reported a negative correlation between dietary omega-3 intake and the incidence of cardiovascular disease, suggesting a preventive potential. Endo and Arita (5) found that omega-3 fatty acids integrate into phospholipid bilayers, thereby influencing membrane fluidity, lipid microdomain formation, and transmembrane signaling, as well as modulating ion channels to prevent arrhythmias. Regarding the nervous system, Heinrichs (6) has shown that omega-3 fatty acids regulate neurotransmission by modifying membrane biophysics and presynaptic vesicle release of amino acid and amine neurotransmitters, thereby enhancing cognitive function and alleviating psychiatric disorders. Collectively, these findings support their use as preventive supplement for cardiovascular and neurological health.

Omega-6 fatty acids, a major subgroup of polyunsaturated fatty acids, primarily include linoleic acid (LA) and arachidonic acid (ARA). Similar to omega-3 fatty acids, they cannot be synthesized endogenously and are mainly obtained from vegetable oils (e.g., corn, soybean, sunflower oil). Omega-6 fatty acids exert diverse biological effects, including anti-inflammatory activity and cardiovascular protection, but their role remains controversial. McCusker et al. reported that omega-6 fatty acids are precursors to pro-inflammatory mediators, such as prostaglandins and leukotrienes. Furthermore, a high omega-6 fatty acid diet may inhibit the anti-inflammatory effects of omega-3 fatty acids. However, whether increased omega-6 or LA intake exacerbates inflammation remains debated. Brouwers et al. (7) and McCusker et al. (8) found that adrenic acid (AdA), an omega-6 fatty acid, inhibits neutrophil production of leukotriene B4 (LTB4) and alleviates inflammation *in vivo*, significantly reducing arthritis symptoms in LTB4-dependent mouse models. Conversely, excessive omega-6 consumption has been associated with heightened inflammation, especially in cardiovascular diseases and certain cancers (9).

Maintaining an optimal omega-6/omega-3 ratio is critical for metabolic and systemic health. Inflammatory markers, including IL-6, CRP, IL-1β, and TNF-α, serve as critical indicators for assessing the body’s inflammatory status. Research has demonstrated that omega-3 and omega-6 fatty acids can modulate the activation and resolution of inflammatory pathways by regulating the expression and secretion of these markers. An excessively high ratio has been shown to attenuate the anti-inflammatory effects of omega-3 fatty acids, and is further associated with increased risk of cardiovascular disease, cancer, and neurological disorders (10–12). Zhang et al. (13) investigated a weak negative correlation between plasma omega-3/6 levels and overall cancer incidence (except prostate cancer). Additionally, Jurado-Fasoli et al. (14) reported that plasma oxidized lipids derived from omega-6 fatty acids (e.g., 15-HeTrE, 5-HETE, 14,15-EpETrE, and the oxidative stress biomarker 8,12-iso-iPF2α-VI) were positively correlated with obesity, metabolic syndrome, fatty liver index, insulin resistance, and lipid parameters in young populations.

In conclusion, the anti-inflammatory effects of omega-3 and omega-6 fatty acids remain debated. While omega-3 fatty acids (e.g., EPA, DHA) are widely recognized as anti-inflammatory, findings regarding omega-6 fatty acids (e.g., ARA), are more heterogeneous. Consequently, the meta-analysis synthesizes high-quality randomized controlled trials (RCTs) to quantitatively compare their anti-inflammatory efficacy across populations and health outcomes, the results provide objective evidence to inform public health strategies and offer direction for future research in fatty acid metabolism and inflammation.

## 2 Materials and methods

### 2.1 Search strategy

This meta-analysis incorporated a comprehensive search across six databases—PubMed, Embase, Cochrane, CNKI, Wanfang, and Weipu—to identify pertinent studies. Adhering rigorously to the PRISMA guidelines, the search strategy employed in all databases included the following terms: ((“Omega-3” OR “ω-3” OR “n-3” OR “Omega 3” OR “eicosapentaenoic acid” OR “docosahexaenoic acid” OR “DHA” OR “EPA”) OR (“Omega-6” OR “ω-6” OR “n-6” OR “Omega 6” OR “linoleic acid” OR “arachidonic acid”) OR ((“Omega-3” OR “ω-3” OR “n-3” OR “Omega 3” OR “eicosapentaenoic acid” OR “docosahexaenoic acid” OR “DHA” OR “EPA”) AND (“Omega-6” OR “ω-6” OR “n-6” OR “Omega 6” OR “linoleic acid” OR “arachidonic acid”))) AND (“Anti-Inflammatory” OR “Anti inflammatory” OR “Anti-Inflammation” OR “inflammation” OR “inflammatory”) AND (“randomized controlled trial” OR “RCT” OR “randomized”).

### 2.2 Inclusion and exclusion criteria

The studies included if they met the following criteria: (1) participants were human subjects; (2) RCTs were employed to maximize the strength of evidence and minimize selection and confounding bias; (3) pregnant or lactating individuals were excluded; (4) trials involving mixed supplementation (e.g., combined omega-3 and omega-6 interventions) or with unspecified dosages were excluded; (5) studies reported quantitative changes in at least one systemic inflammatory marker; (6) sufficient data were available, including extractable mean ± standard deviation (or standard error), sample size, *p*-value, and other relevant statistics. Studies that only presented graphical results, lacked specific values, or had more than 20% missing data were excluded.

The exclusion criteria were as follows: (1) intervention arms using anti-inflammatory medications, such as antibiotics, glucocorticoids, growth hormone, etc.; (2) concomitant use of drugs affecting fatty acid metabolism, such as lipid-lowering drugs (fibrates, statins), drugs that affect fatty acid absorption and transport (orlistat, valproic acid), etc. (3) animal or *in vitro* studies.

### 2.3 Data extraction and quality assessment

This study was conducted in accordance with the PRISMA guidelines (15). All eligible articles were assessed based on the predefined PICOS criteria. Two researchers independently reviewed the search results using the established terms and screened studies by title, keywords, abstract, and full text, in line with the inclusion and exclusion criteria. Duplicate and irrelevant records were first removed, followed by the exclusion of studies with ambiguous data, missing values, or inconsistent outcome measures. Data extraction was performed by one researcher and cross-verified by another. Extracted information included study characteristics (e.g., design, population, interventions, comparators, and outcomes), group allocation, sample size, and outcome measures, which were systematically tabulated. When required data were not explicitly reported, the original authors were contacted for clarification. The methodological quality of the included studies was evaluated using the Cochrane Risk of Bias Assessment Tool, focusing on five specific domains: randomization process, adherence to interventions and blinding, completeness of outcome data, objectivity of outcome measurement, and selective reporting.

### 2.4 Data synthesis and statistical analysis

Extracted data were expressed as the mean ± standard deviation (SD). When original studies reported outcomes in alternative formats, values were converted to mean ± SD using established statistical methods or in accordance with the Cochrane Handbook (16). Statistical heterogeneity across studies was assessed using the *I*^2^ statistic. An *I*^2^ ≤ 25% was considered indicative of low heterogeneity, and a fixed-effects model was applied. An *I*^2^ between 25 and 50% indicated moderate heterogeneity, while *I*^2^ > 50% denoted substantial heterogeneity, for which a random-effects model was employed. In cases of high heterogeneity, sensitivity analyses were performed by sequentially excluding studies with potential bias. Subgroup analyses were conducted to explore potential sources of heterogeneity and to identify confounding factors, with the aim of reducing *I*^2^ values within subgroups. The presence of reporting bias was assessed, with statistical significance set at *p* < 0.05. Given that fewer than 10 studies were included, funnel plot bias analysis was not performed.

## 3 Results

### 3.1 Literature search results

**Figure 1:**
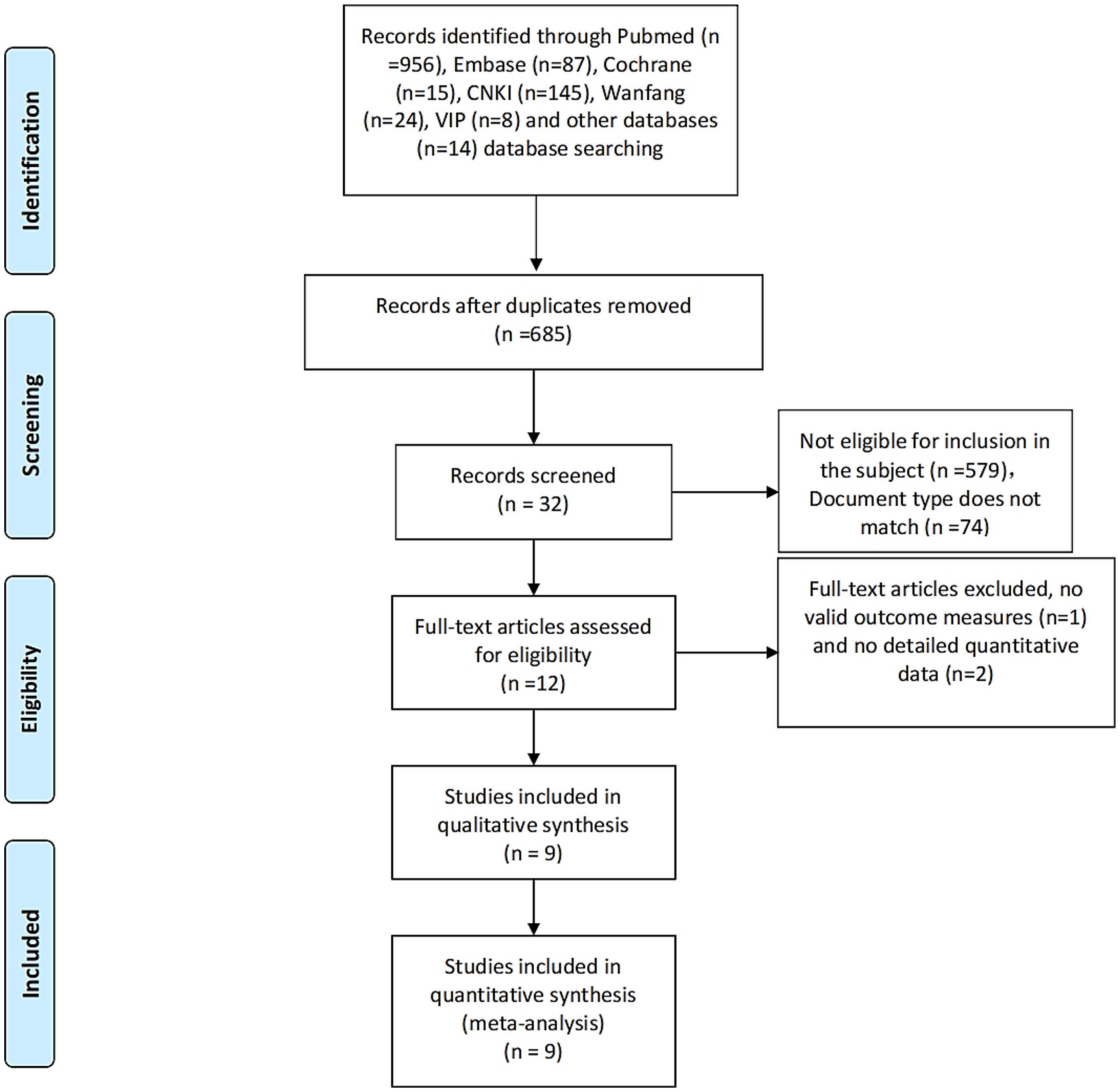
Retrieval flow chart.

The study selection process is summarized in the PRISMA flow diagram (Figure 1). Initially, a total of 1,249 records were initially retrieved, of which 685 articles remained after duplicate removal. Screening of titles, abstracts, and article types led to the exclusion of 653 records. Following full-text assessment, 12 articles met the predefined inclusion criteria. Of these, 9 RCTs with complete data and relevant outcome indicators were included in the final analysis.

### 3.2 Characteristics and quality assessment of included studies

**Figure 2:**
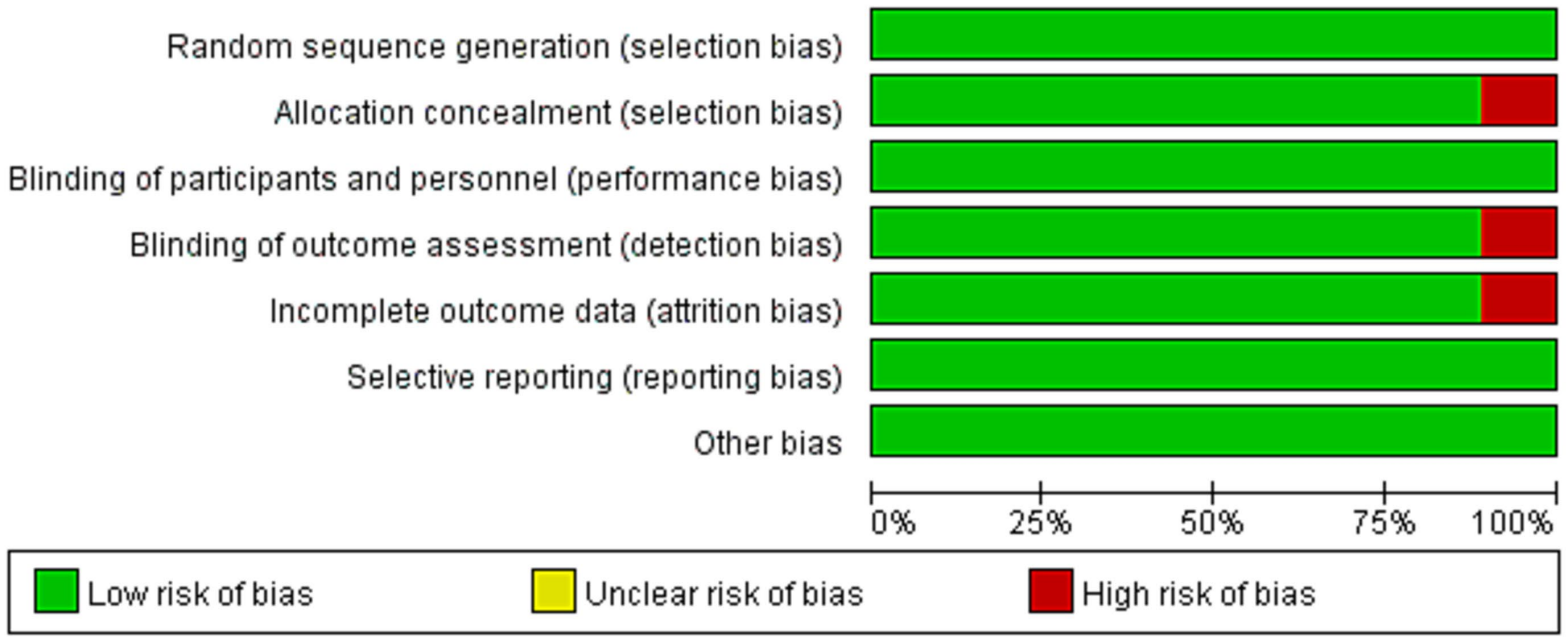
Cochrane bias risk assessment-1.

**Figure 3:**
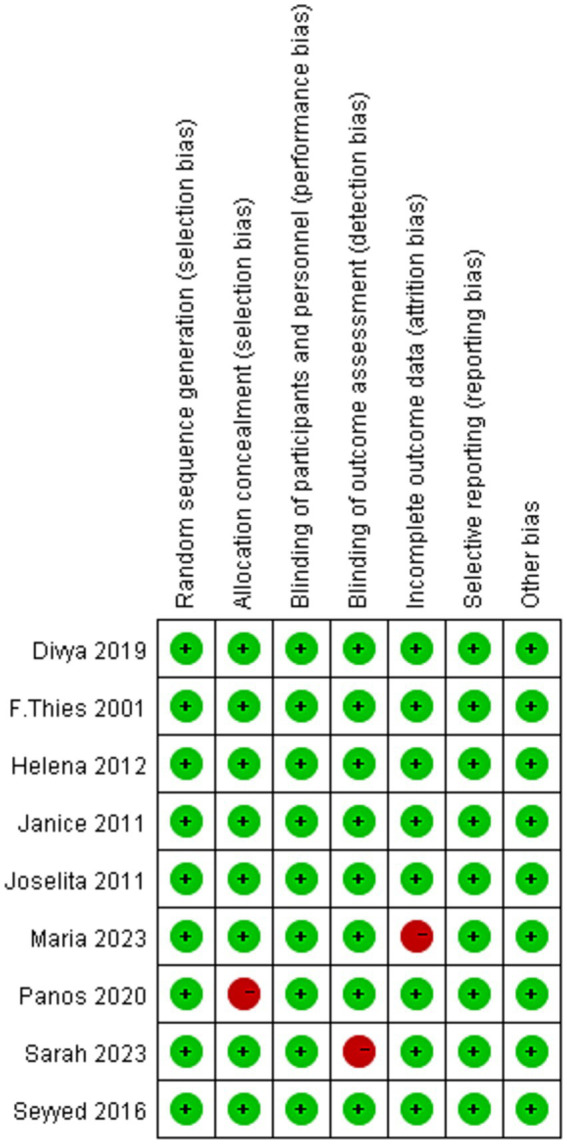
Cochrane bias risk assessment-2.

The baseline characteristics of the nine included RCTs (17–19) are summarized in Table 1. In total, 504 participants were enrolled, comprising 254 in the intervention groups and 250 in the placebo group. The trials were published between 2011 and 2023, with the majority conducted in Europe and North America: six in the United States and one each in Greece, Spain, Iran, and the United Kingdom. Four studies investigated healthy populations (Birudaraju, Thies, Siqueira, and Kiecolt-Glaser et al.), whereas the remaining five targeted patients with specific diseases. The sample sizes ranged from 7 to 38 per group (median: 30), with 44.4% (4/9) enrolling ≤20 participants. Notably, the trial by Thies et al. included two independent intervention arms (omega-3 and omega-6). Risk of bias was assessed using the Cochrane tool (Figures 2, 3), and overall, the included RCTs were judged to have a low risk of bias.

**Table 1:** Characteristics of included articles.

No.	Author (year, country)	Design	Group	Participants	Intervention and comparison	Dose	Duration (weeks/days)
1	Chiu et al. (20) (2012, USA)	RCT	Omega 3 + 6 = 38 Placebo = 38	People with autism	EPA + DHA + GLA vs. placebo (canola oil + lemon flavor, matched in appearance/taste)	25–100 mg/kg	90 days
2	Innes et al. (21) (2018, USA)	RCT	Omega 3 + 6 = 29 Placebo = 30	Healthy people	EPA + DHA + GLA + curcumin vs. placebo (identical softgels with canola oil)	Fixed dose (4 capsules per day)	4 weeks
3	Thies et al. (17) (2001, UK)	RCT	Omega 3 = 7 Placebo = 8 and Omega 6 = 7 Placebo = 8	Healthy people	FO (EPA + DHA) vs. placebo oil (palm oil:sunflower oil = 80:20) and GLA vs. placebo oil (palm oil:sunflower oil = 80:20)	FO: 1 g (EPA 720 mg + DHA 280 mg) and GLA: ~700 mg target fatty acids	12 weeks
4	Bjermo et al. (18) (2012, USA)	RCT	Omega 6 = 32 Placebo = 29	Obese people	Omega 6 PUFA (linoleic acid, mainly from sunflower oil, margarine, sunflower seeds, etc.) vs. SFA (mainly from butter)	About 40 g/day	10 weeks
5	Singh et al. (22) (2024, USA)	RCT	Omega 6 = 12 Placebo = 12	Healthy people	Soybean oil-based fat emulsion (Intralipid^®^, pure soybean oil) versus saline control	1.5 g/kg/day	24 h
6	Yang et al. (23) (2019, Greece)	RCT	Omega 3 = 30 Placebo = 29	Premature babies	MCT/ω-3 blend emulsion (Smoflipid^®^) vs. Soybean oil-based emulsion (Intralipid^®^)	Initial 1 g/kg/day → Maximum 3 g/kg/day	15 days
7	Quetglas-Llabrés et al. (19) (2023, Spain)	RCT	Omega 3 = 35 Placebo = 32	Nonalcoholic fatty liver disease patients	High adherence Mediterranean diet (olive oil) vs. low adherence Mediterranean diet	7 meals a day	12 months
8	Wang et al. (24) (2019, Iran)	RCT	Omega 3 = 30 Placebo = 30	Nephritis patients	Omega-3 supplement (flaxseed oil) vs. placebo (ingredients not specified, matched appearance)	1,000 mg/day	12 weeks
9	Dong et al. (25) (2017, USA)	RCT	Omega 3 + 6 = 34 Placebo = 34	Healthy people	Omega-3 supplements (fish oil: EPA + DHA) vs. placebo: mixed vegetable oils (palm oil, olive oil, soybean oil, etc.)	2.5 g/ten (EPA 2,085 mg + DHA 348 mg)	12 weeks

EPA, eicosapentaenoic acid; DHA, docosahexaenoic acid; GLA, gamma-linolenic acid; FO, fish oil; RCT, randomized controlled trial PUFA, polyunsaturated fatty acids; SFA, saturated fatty acids; MCT, medium-chain triglycerides.

### 3.3 Main results of subgroup analysis

#### 3.3.1 Subgroup analysis by omega-3 and omega-6 classification

**Figure 4:**
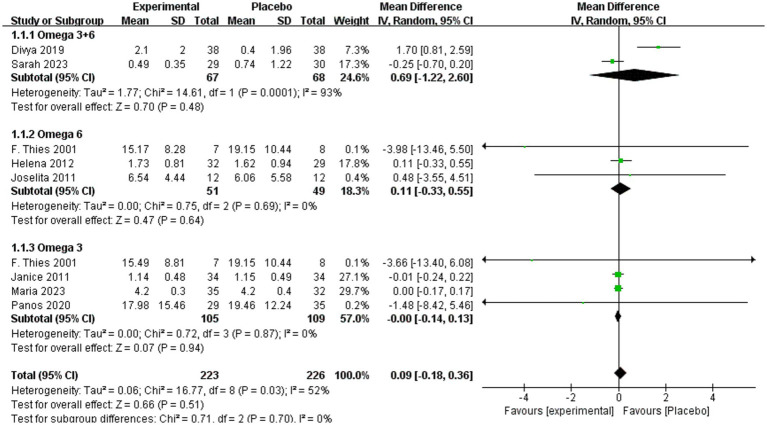
Forest plot of IL-6 subgroup analysis.

A subgroup analysis was conducted according to fatty acids (omega-3 + 6, omega-3, and omega-6), with outcomes including IL-6, CRP, IL-1β, and TNF-α. As shown in Figure 4, no significant differences in IL-6 levels were observed between intervention and placebo groups overall (MD = 0.09, 95% CI: −0.18 to 0.36, *p* = 0.51, *I*^2^ = 52%). In subgroup analyses, IL-6 levels did not differ significantly between omega-3 + 6 and placebo (MD = 0.69, 95% CI: −1.22 to 2.60, *p* = 0.48, *I*^2^ = 93%), omega-6 and placebo (MD = 0.11, 95% CI: −0.33 to 0.55, *p* = 0.64, *I*^2^ = 0%) or omega-3 and placebo (MD = −0.00, 95% CI: −0.14 to 0.13, *p* = 0.51, *I*^2^ = 0%).

**Figure 5:**
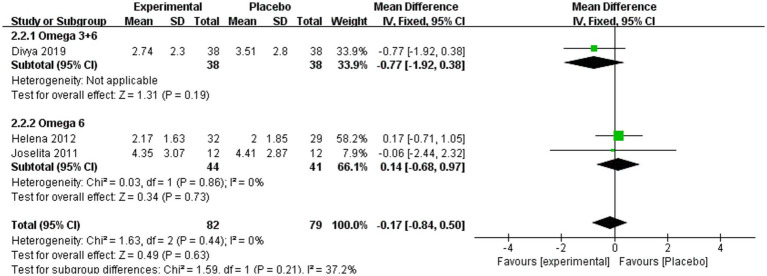
Forest plot of CRP subgroup analysis.

Figure 5 shows that CRP levels also did not differ significantly overall (MD = −0.17, 95% CI: −0.84 to 0.50, *p* = 0.63, *I*^2^ = 0%). No significant differences were observed between omega-3 + 6 and the placebo (MD = −0.77, 95% CI: −1.92 to 0.38, *p* = 0.19) or omega-6 and placebo (MD = 0.14, 95% CI: −0.68 to 0.97, *p* = 0.73, *I*^2^ = 0%).

**Figure 6:**
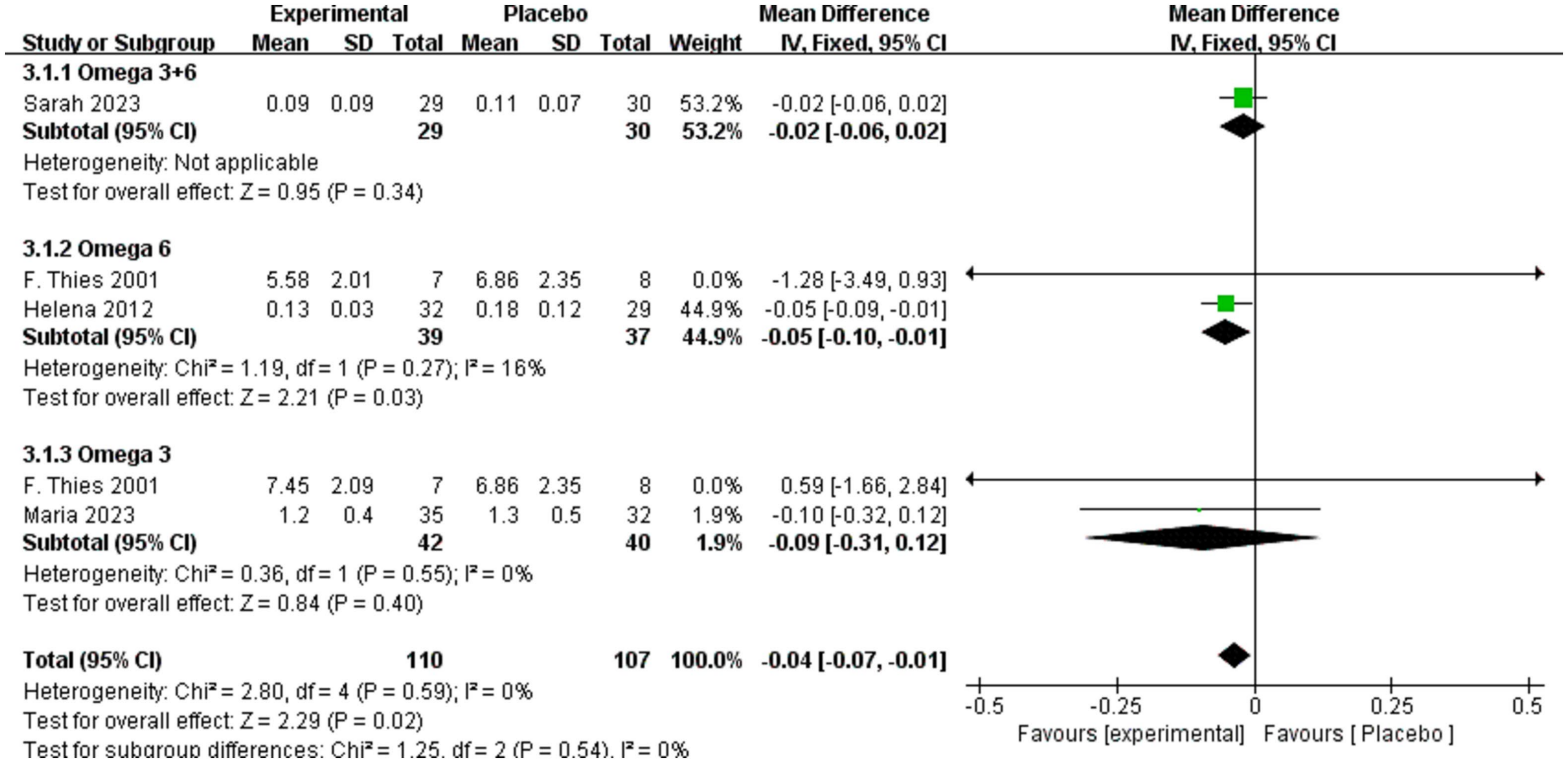
Forest plot of IL-1β subgroup analysis.

By contrast, Figure 6 indicates that long-term omega-3/6 supplementation was associated with a significant reduction of IL-1β compared with placebo (MD = −0.04, 95% CI: −0.07 to −0.12, *p* = 0.02, *I*^2^ = 0%). Subgroup analyses revealed no significant effect for omega-3 + 6 (MD = 0.02, 95% CI: −0.06 to 0.02, *p* = 0.34) or omega-3 (MD = −0.09, 95% CI: −0.31 to 0.12, *p* = 0.40, *I*^2^ = 0%). However, omega-6 supplementation significantly reduced IL-1β levels compared with placebo (MD = −0.05, 95% CI: −0.10 to −0.01, *p* = 0.03, *I*^2^ = 16%). In the four studies included, IL-1β was consistently measured in pg/mL. In the study by Sarah et al., the baseline IL-1β levels were 0.11 pg/mL in the omega 3–6 treatment group and 0.10 pg/mL in the placebo group. Thies et al. reported a detection limit of 2 pg/mL in their methodology but did not specify the baseline IL-1β values for each group in their results. Similarly, Helena et al. identified IL-1β as a detection indicator but did not provide specific baseline values in the results section. In the study by Maria et al., which examined adherence to the Mediterranean diet, the baseline IL-1β levels were 1.3 pg/mL in the low-adherence group and 1.2 pg/mL in the high-adherence group.

**Figure 7:**
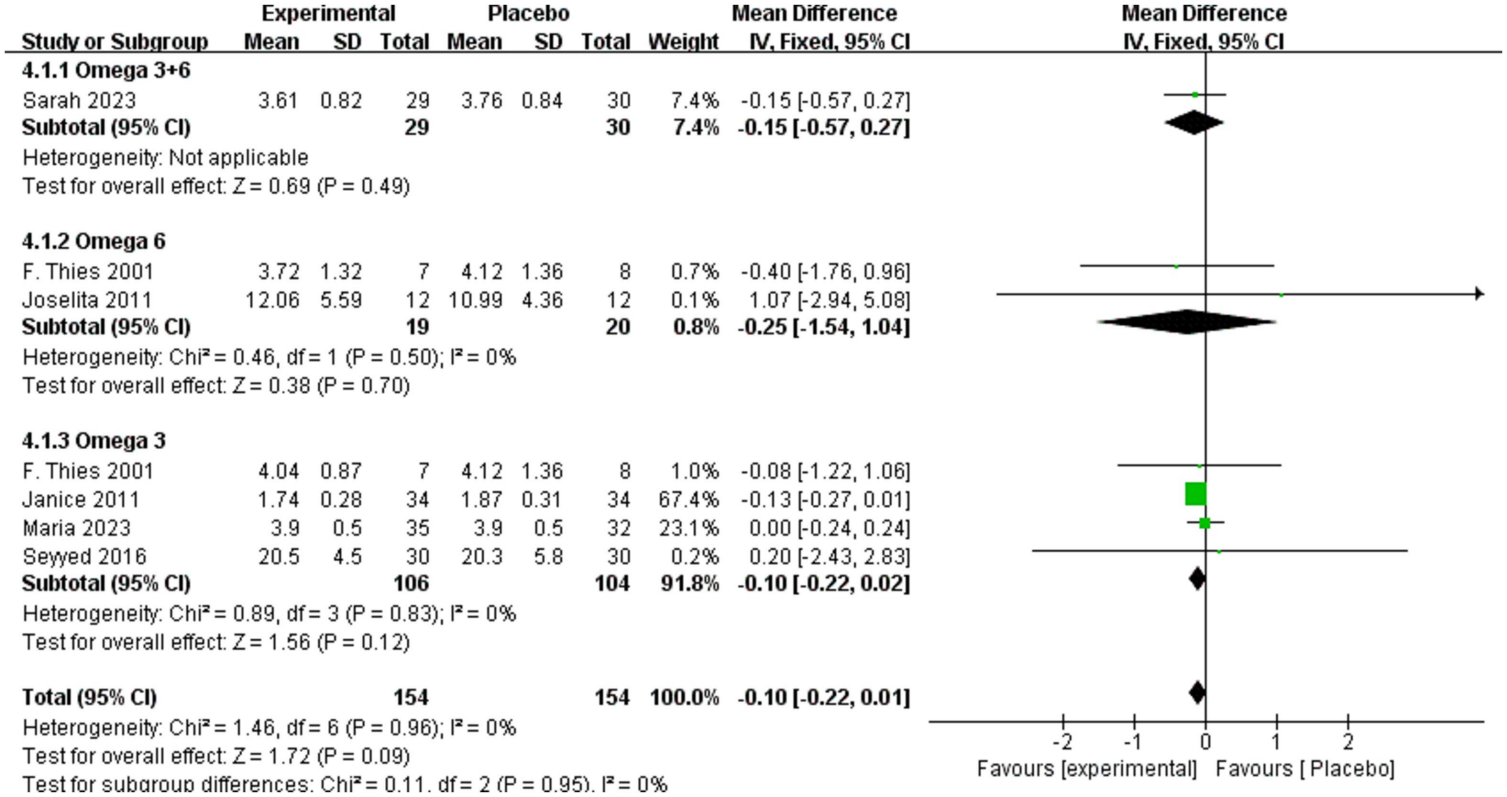
Forest plot of TNF-α subgroup analysis.

As shown in Figure 7, TNF-α levels did not differ significantly between intervention and placebo groups overall (MD = 0.10, 95% CI: −0.22 to 0.01, *p* = 0.09, *I*^2^ = 0%). Subgroup analyses confirmed no significant differences for omega-3 + 6 (MD = −0.15, 95% CI: −0.57 to 0.27, *p* = 0.49), omega-6 (MD = −0.25, 95% CI: −1.54 to 1.04, *p* = 0.70, *I*^2^ = 0%) or omega-3 (MD = −0.10, 95% CI: −0.22 to 0.02, *p* = 0.12, *I*^2^ = 0%).

#### 3.3.2 Subgroup analysis of healthy and patient populations

**Figure 8:**
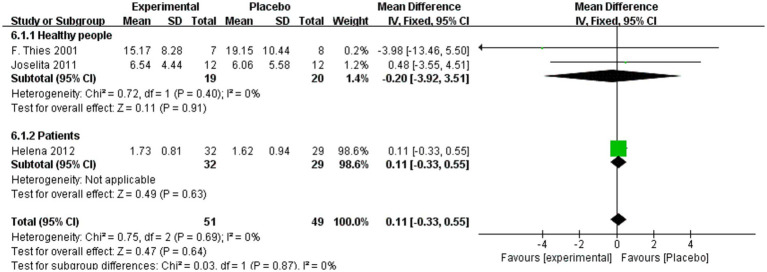
Analysis of IL-6 + omega-6 in healthy and patient subgroups.

In this subgroup analysis, the “patient population” primarily comprises individuals diagnosed with MASLD, obesity, kidney diseases such as diabetic nephropathy, and autism, among others, all of whom have well-defined disease diagnoses. These conditions are characterized by varying levels of chronic inflammation, providing a suitable context for the preliminary investigation of the differential anti-inflammatory effects of omega-3 and omega-6 fatty acids under pathological conditions. A subgroup analysis was conducted to evaluate IL-6 levels in healthy individuals and patients. As shown in Figure 8, no significant differences in IL-6 levels were observed between intervention and placebo groups among participants consuming omega-6 (MD = 0.11, 95% CI: −0.33 to 0.55, *p* = 0.64, *I*^2^ = 0%). In subgroup analyses, IL-6 levels did not differ significantly in either healthy individuals (MD = −0.20, 95% CI: −3.92 to 3.51, *p* = 0.91, *I*^2^ = 0%) or patients with obesity (MD = 0.11, 95% CI: −0.33 to 0.55, *p* = 0.63).

**Figure 9:**
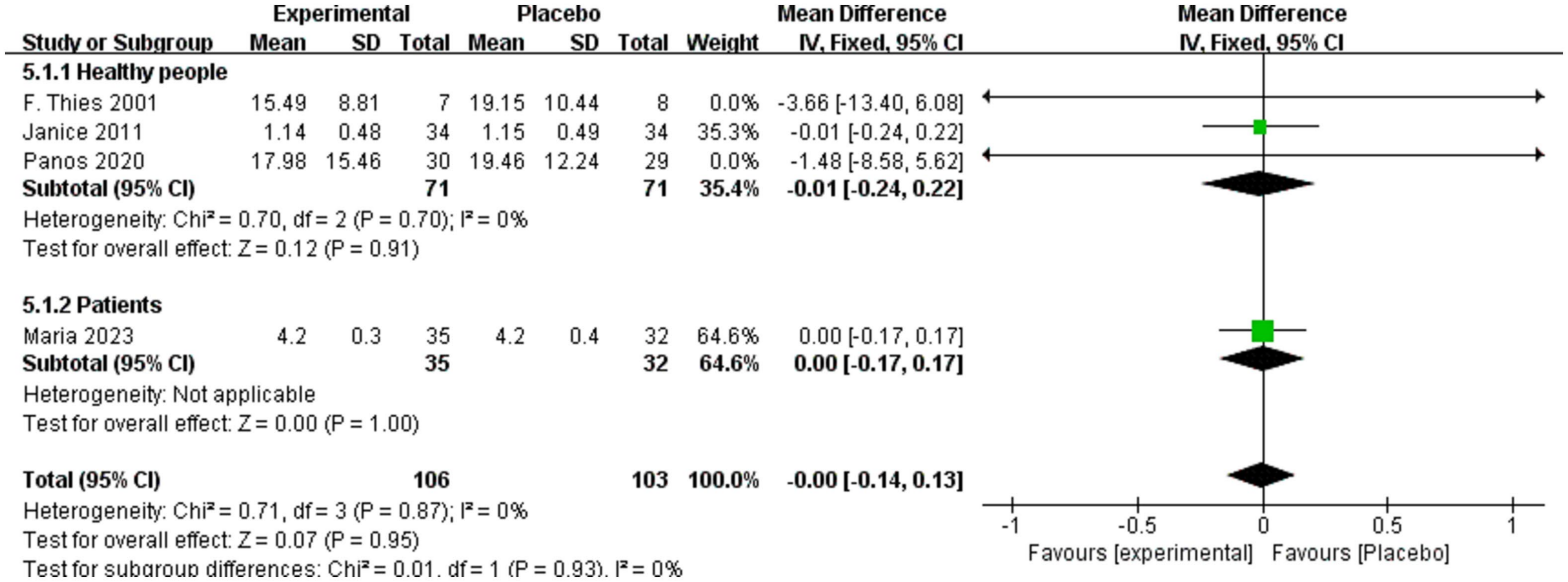
Analysis of IL-6 + omega-3 in healthy and patient subgroups.

Similarly, Figure 9 presents no significant differences in IL-6 levels between intervention and placebo groups among participants consuming omega-3 (MD = 0.00, 95% CI: −0.14 to 0.13, *p* = 0.95, *I*^2^ = 0%). Subgroup analyses confirmed no significant effects in either healthy individuals (MD = −0.01, 95% CI: −0.24 to 0.22, *p* = 0.91, *I*^2^ = 0%) or patients with non-alcoholic fatty liver disease (MD = 0.00, 95% CI: −0.17 to 0.17, *p* = 1.00).

#### 3.3.3 Results of 10–12 weeks of omega consumption

**Figure 10:**
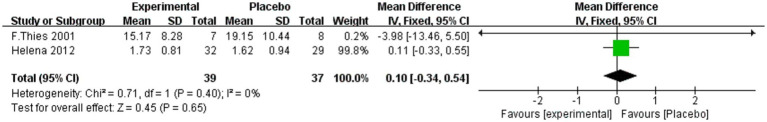
Forest plot of IL-6 results of omega-6.

**Figure 11:**
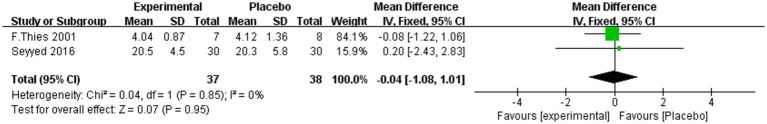
Forest plot of TNF-α analysis results of omega-3.

To reduce potential bias and heterogeneity arising from differences in intervention duration, a subgroup analysis was performed including only studies with a consumption period of 10–12 weeks. As shown in Figure 9, omega-6 intake for 10–12 weeks did not result in a significant difference in IL-6 levels between intervention and placebo groups (MD = 0.10, 95% CI: −0.34 to 0.54, *p* = 0.65, *I*^2^ = 0%) (Figure 9). Similarly, omega-3 supplementation for 10–12 weeks showed no significant effect on TNF-α levels compared with placebo (MD = −0.04, 95% CI: −1.08 to 1.01, *p* = 0.95, *I*^2^ = 0%) (see Figures 10, 11).

To address the issue of multiple testing across the four primary inflammatory markers (IL-6, CRP, IL-1β, and TNF-α), we utilized the Benjamini–Hochberg false discovery rate (FDR) procedure, applying a *Q*-value threshold of 0.10. This approach effectively controls the expected proportion of false positives among the significant findings and is widely recognized as appropriate for exploratory meta-analyses. The revised results section presents both the FDR-adjusted *Q*-values and the unadjusted *p*-values, as illustrated in Table 2. The final outcomes corroborate previous research, affirming the statistical significance of IL-1β.

**Table 2:** Benjamini–Hochberg false discovery rate (FDR) for meta-analysis outcome.

Ranking	Outcome	*p*-value	Critical value (*k*/4 × 0.10)	Significance (*p* ≤ critical value)
1	IL-1β	0.02	(1/4) × 0.10 = 0.025	Significant
2	TNF-α	0.09	(2/4) × 0.10 = 0.050	Not significant
3	IL-6	0.51	(3/4) × 0.10 = 0.075	Not significant
4	CRP	0.63	(4/4) 0.10 = 0.100	Not significant

As indicated by the GRADE systematic reviews presented in Table 3, the quality of evidence for all outcome measures, including IL-6, CRP, IL-1β, and TNF-α, in this meta-analysis ranged from moderate to high. The primary factors contributing to the downgrading of evidence quality were risk of bias, stemming from limitations in study design, and imprecision, due to small sample sizes and uncertain effect sizes. Consequently, future research with larger sample sizes and enhanced methodological rigor is necessary to substantiate the potential anti-inflammatory effects of omega fatty acids.

**Table 3:** GRADE evidence quality assessment form.

Outcome measures	Number of studies and study design	Risk of bias	Inconsistency	Indirectness	Imprecision	Publication bias	Quality of evidence
IL-6	8 RCTs	Not serious	Not serious	Not serious	Serious	None	Middle
CRP	3 RCTs	Serious	Not serious	Not serious	Not serious	None	Middle
IL-1β	5 RCTs	Serious	Not serious	Not serious	Not serious	None	Middle
TNF-α	6 RCTs	Not serious	Not serious	Not serious	Not serious	None	High

## 4 Discussion

This study represents the first meta-analysis to directly compare the effects of omega-3 + 6, omega-3, and omega-6 unsaturated fatty acids on anti-inflammatory marker levels in humans. Overall, the pooled results indicated no significant improvements in IL-6 (MD = 0.09, *p* = 0.51), CRP (MD = −0.17, *p* = 0.63), and TNF-α (MD = 0.10, *p* = 0.09) compared with placebo. However, a notable finding was that supplementation with omega-3 or omega-6 significantly reduced IL-1β levels (MD = −0.04, *p* = 0.02), with the effect being more pronounced in the omega-6 subgroup (MD = −0.05, *p* = 0.03). In contrast, omega-3 intervention alone did not significantly lower IL-1β (MD = −0.09, *p* = 0.40), which may reflect limitations in dosage, participant numbers, or intervention duration. The observed anti-inflammatory role of omega-6 acids aligns with recent mechanistic studies. Possible reasons include derivatives such as 17-hydroxyeicosapentaenoic acid (17-HDPAn-6) and 10,17-dihydroxyeicosapentaenoic acid (10,17-HDPAn-6) have been shown to modulate macrophage polarization toward the M2 phenotype, thereby enhancing phagocytosis and suppressing TNF-α and iNOS expression while upregulating IL-1 receptor antagonist and scavenger receptor expression (20). Therefore we infer this polarization results in the downregulation of TNF-α and inducible nitric oxide synthase gene expression, while upregulating the expression of chemokine IL-1 receptor antagonist and type A scavenger receptor. Intraperitoneal administration in mice demonstrated the potential to mitigate inflammatory conditions, such as inflammatory bowel disease. Traditionally, omega-6 fatty acids, particularly ARA, are regarded as pro-inflammatory due to their role as precursors to prostaglandins and leukotrienes (21). The present findings challenge this one-dimensional view by suggesting that omega-6 fatty acids can exhibit both pro-inflammatory and, under certain circumstances, anti-inflammatory effects. Nonetheless, the study did not further explore the anti-inflammatory effects of omega-6 fatty acids across varying doses due to a lack of data uniformity. The complexity of this issue may be intricately linked to dosage variations and differing metabolic environmental conditions. In this study, a comparative analysis between healthy individuals and patients, as well as a subgroup analysis of healthy and obese individuals, revealed that IL-6 levels did not exhibit significant changes following omega-3 or omega-6 interventions. Furthermore, the subgroup analysis of patients with MASLD demonstrated that omega-3 intervention did not result in any improvement in IL-6 levels (MD = 0.00, *p* = 1.00). This finding contradicts the prevailing conclusions of current mainstream studies, which suggest that omega-3 can significantly ameliorate liver insulin resistance and hepatic steatosis (3, 22–25).

The discrepancy may be attributed to two primary factors: first, patients with MASLD experience long-term chronic inflammation, which could lead to tolerance, making it difficult for a single omega-3 fatty acid intervention to reverse the established inflammatory environment. Second, the small sample size (*n* = 35) introduces potential bias, and may not be sufficient to fully support the conclusion. In healthy individuals, omega-3 and omega-6 interventions do not seem to significantly affect IL-6 and TNF-α levels. It is speculated that omega-3 or 6 may play a role in physiological conditions mainly by preventing the regulation of macro-metabolism rather than directly inhibiting the release of inflammatory factors. In addition, the results showed that 10–12 weeks of intervention failed to change the levels of IL-6 (omega-6 group) or TNF-α (omega-3 group). In general, omega-3 (e.g., EPA/DHA) and omega-6 fatty acids (e.g., adrenic acid) compete for the cyclooxygenase (COX) and lipoxygenase (LOX) pathways, yet they yield distinct physiological functions. Prior research has demonstrated that the omega-6 derivative ARA serves as a precursor to proinflammatory mediators, including prostaglandins and leukotrienes, which facilitate the body’s inflammatory defense mechanisms by promoting inflammatory responses (19). Concerning the anti-inflammatory regulatory effects of omega-6 fatty acids, prevailing theories suggest that the omega-6 polyunsaturated fatty acid derivative, adrenic acid, exhibits the capacity to inhibit leukotriene B4 production in specific inflammatory models (7). Furthermore, metabolites of omega-6 fatty acids, such as lipoxins, have been identified to possess both anti-inflammatory and pro-inflammatory properties. These metabolites contribute to the resolution of inflammation by diminishing the recruitment of inflammatory cells and enhancing the phagocytosis of apoptotic cells by macrophages. Additionally, the interaction between omega-6 and omega-3 fatty acids can, in certain contexts, influence the body’s inflammatory response (21). While this study controls for errors arising from other dietary interventions, it is imperative to account for the broader variations in regional diets. Western diets typically exhibit a high omega-6/omega-3 ratio, ranging from 10:1 to 20–25:1, in contrast to the approximately 1:1 ratio observed in ancestral human diets (26). Due to the limited number of studies and heterogeneity in the types of omega-6 fatty acids used (e.g., LA vs. GLA), a more granular subgroup analysis was not feasible. This heterogeneity prevents us from drawing definitive conclusions about structure-specific effects.

Consequently, the inclusion of participants from this region may have attenuated the anti-inflammatory effects of omega-3 in the study’s findings. The study also identified that omega-6 fatty acids can reduce IL-1β levels; however, this effect should be considered context-specific, and omega-6 fatty acids should not be categorically labeled as “pro-inflammatory.” Their function likely involves an interplay of factors, including the metabolic environment, the presence of omega-3 fatty acids, derivative types, and dosage. Although the study reported negative outcomes for omega-3 fatty acids, it is important to note that the duration of most included studies did not exceed 1 year. Therefore, the potential long-term benefits of omega-3 fatty acids should not be dismissed. To further evaluate the clinical significance of the observed effect size, we conducted a comprehensive review of the existing literature and determined that a minimum clinically important difference (MCID) criterion for IL-1β has not been established in comparable populations. This finding is not unexpected, as the threshold for clinical significance of IL-1β, an exploratory inflammatory marker, is generally less well-defined than that for IL-6 or C-reactive protein (CRP). Consequently, we are unable to directly compare the effect size observed in this study with established MCIDs. Importantly, other key inflammatory markers, such as IL-6, CRP, and TNF-α, did not exhibit significant changes following the intervention. This isolated positive result suggests that the reduction in IL-1β should be interpreted with caution. In light of these considerations, we propose that the effect of omega-6 fatty acids on IL-1β should currently be regarded as an exploratory finding rather than a definitive clinical benefit. Future research involving larger sample sizes and focusing on inflammation-related clinical events as primary endpoints is necessary to validate the clinical relevance of this observation and to investigate its underlying mechanisms. The observed reduction in IL-1β in this study was 0.06 pg/mL (95% CI: −0.07 to −0.01), indicating a relatively minor absolute effect size. Given the absence of an established minimum clinically important difference (MCID) for IL-1β, the clinical significance of this change remains indeterminate. Importantly, unlike IL-1β, other inflammatory markers such as IL-6 and CRP did not exhibit significant alterations following the intervention, suggesting that this isolated effect warrants cautious interpretation. Consequently, we propose that the impact of omega-6 fatty acids on IL-1β should presently be regarded as an exploratory finding rather than a definitive clinical benefit. Future research involving larger sample sizes and focusing on inflammation-related clinical events as primary endpoints is essential to ascertain the clinical relevance of this observation.

The majority of the RCTs incorporated in this study utilized placebo formulations containing PUFAs, such as rapeseed oil and mixed vegetable oils, to mimic typical dietary habits within the control group. Although this design may obscure the net effect of specific ω-fatty acids in the intervention group and impede the precise evaluation of the independent anti-inflammatory effects of ω-3 and ω-6 fatty acids, the complete exclusion of normal dietary fats is not ethically justifiable. Consequently, it is essential to incorporate such trials to preserve sample size and ensure representativeness. Future research incorporating control designs with neutral carriers, such as mineral oil or starch-based substances, would be more effective in isolating the specific effects of PUFAs. The RCTs included in this study were conducted across diverse global regions, including the United States, Europe, and Asia, among others. Consequently, there were notable variations in baseline omega fatty acid levels, intervention dosages, and treatment durations across the studies. Furthermore, the reporting standards for indicators related to fatty acid metabolism differ between countries and regions, complicating the systematic evaluation of these factors’ impacts on tissue accumulation and effect accumulation within the current analysis. We recognize this as a significant limitation of our exploratory study. As future RCTs with standardized dosing and baseline assessments become available, subgroup analyses and discussions regarding dose-effect relationships will likely become more comprehensive. While this study identified a correlation between omega-6 fatty acids and the reduction of IL-1β, it did not directly compare the differential effects of omega-3 and ω-6 fatty acids at equivalent doses within the same population. Furthermore, the current data do not substantiate a causal relationship. The anti-inflammatory effects of omega-6 fatty acids may be contingent upon a particular metabolic environment or the resultant oxylipin profile. Future research should include head-to-head randomized controlled trials and the incorporation of metabolomics data to systematically elucidate the mechanisms of action and potential interaction effects of various PUFAs.

Furthermore, in future research, it is imperative to conduct subgroup analyses across a broader spectrum of disease populations, particularly those with elevated cardiovascular risk. This is due to the potential beneficial effects of omega-3 fatty acids on cardiovascular risk factors, such as reducing triglycerides, exerting anti-inflammatory effects, and enhancing metabolic processes (27–33). Individuals with low baseline levels of omega-3 may derive greater benefits. However, this study has several limitations. Primarily, the small sample size limits the statistical power, increasing the likelihood of false negatives due to sample bias. Additionally, small-sample RCTs are prone to publication bias favoring positive results. When the number of included studies is 10 or fewer, funnel plots cannot be effectively employed, potentially overlooking negative outcomes. Furthermore, the long half-life of certain biomarkers, such as CRP at 19 h, poses a challenge for short-term interventions (e.g., Siqueira’s 24-h study) to accurately capture changes. In addition, the results of this study on omega-6 have limited interpretability, and future research needs to conduct further meta-analyses to compare different types of omega-6. Finally, some placebos use vegetable oils with a composition similar to dietary fatty acids, which are not completely inert and may weaken the real effect difference between the intervention group and the control group, which is one of the limitations of the study.

## 5 Conclusion

This meta-analysis demonstrates that omega-6 fatty acids possess potential anti-inflammatory properties, significantly reducing IL-1β levels, thereby challenging the conventional view of omega-6 fatty acids as predominantly pro-inflammatory. Conversely, the findings indicate that omega-3 fatty acids do not exert a significant impact on IL-6, CRP, or TNF-α. Future research should focus on a comprehensive evaluation of these effects by integrating individual factors and diverse populations across various regions and dosages. Additionally, increasing the sample size is essential to validate the specific anti-inflammatory potential of distinct omega fatty acids and to enhance the development of nutritional intervention strategies.

## Data Availability

The original contributions presented in the study are included in the article/supplementary material, further inquiries can be directed to the corresponding author/s.
